# Evaluation of a High-Temperature Pre-Heating System Design for a Large-Scale Additive Manufacturing System

**DOI:** 10.3390/mi13091475

**Published:** 2022-09-05

**Authors:** Rabelani Duncan Ramulifho, Kapil Gupta, Daniel Glaser

**Affiliations:** 1 Department of Mechanical and Industrial Engineering Technology, University of Johannesburg, Johannesburg 2028, South Africa; 2 Photonics Centre, Council for Scientific and Industrial Research, Pretoria 0001, South Africa

**Keywords:** additive manufacturing, high-temperature insulation, residual stress, selective laser melting, thermal analysis

## Abstract

Additive Manufacturing (AM) of titanium (Ti6Al4V) material using Selective Laser Melting (SLM) may generate significant residual stresses of a tensile nature, which can cause premature component failure. The Aeroswift platform is a large volume AM machine where a high-temperature substrate preheating system is used to mitigate high thermal gradients. The current machine platform is unable to achieve a target build-plate temperature of 600 °C. This study focuses on the analysis of the preheating system design to determine the cause of its inefficiency, and the experimental testing of key components such as the heater and insulation materials. A Finite Element Analysis (FEA) model shows the ceramic heater achieves a maximum temperature of 395 °C, while the substrates (build-plates) only attain 374 °C. Analysis showed that having several metal components in contact and inadequate insulation around the heater caused heat loss, resulting in the preheating system’s inefficiency. Additionally, experimental testing shows that the insulation material used was 44% efficient, and a simple insulated test setup was only able to obtain a maximum temperature of 548.8 °C on a 20 mm thick stainless steel 304 plate, which illustrated some of the challenges faced by the current pre-heating design. New design options have been developed and FEA analysis indicates that a reduction in heat loss through improved sub-component configurations can obtain 650 °C degrees above the substrate without changing the heating element power. The development and challenges associated with the large-scale preheating system for AM are discussed, giving an insight into improving its performance.

## 1. Introduction

**Figure 1:**
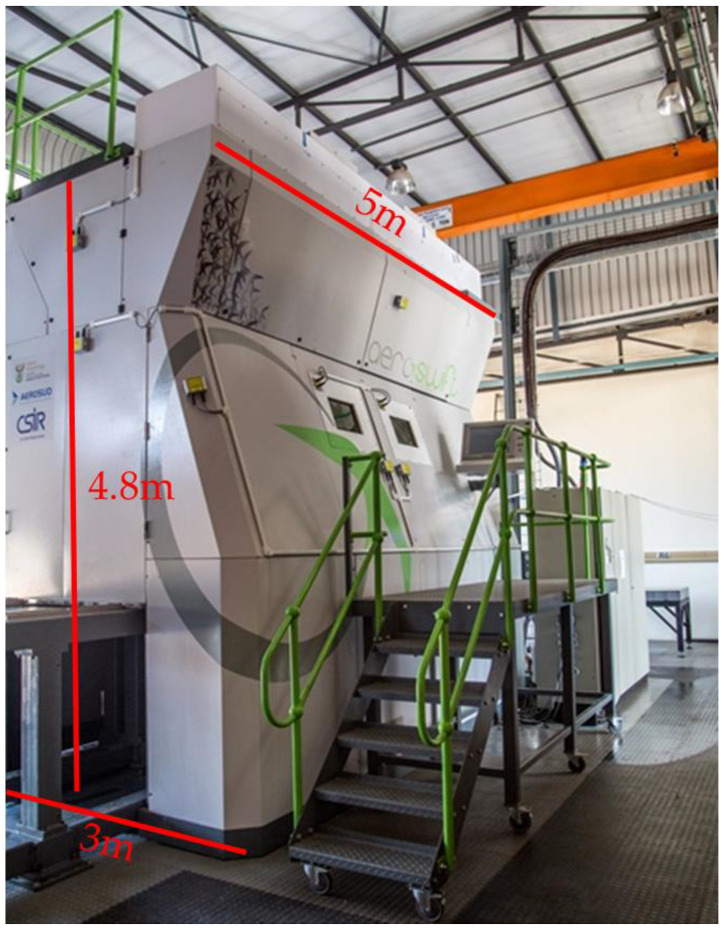
Aeroswift 3D printing machine.

Additive Manufacturing (AM) is a promising technological process that fabricates a full 3D component layer-by-layer directly from a 3D model, rather than using common manufacturing methods (machining) that remove material [1,2]. AM enables engineers to design parts with intricate shapes that meet client requirements in accordance with their imaginations [2]. The aerospace and automotive industry can use AM to produce lightweight components that can lower the amount of fuel consumed [3]. Machines with larger build volumes are capable of fabricating large quantities of parts with distinct shapes at once [1]. Selective Laser Melting (SLM) is a Laser Powder Bed Fusion (LPBF) technique that selectively melts powder layers using a laser to fabricate a 3D component according to the cross-sectional profile of the Computer-Aided Drafting (CAD) model [4,5]. Aeroswift (see Figure 1) is a large volume AM machine designed and constructed in South Africa (Pretoria) in a collaboration between the Aerospace company Aerosud and the Council for Scientific and Industrial Research (CSIR). The Aeroswift uses SLM technology to build complex titanium parts using a fiber laser of up to 5 kW output power. 

**Figure 2:**
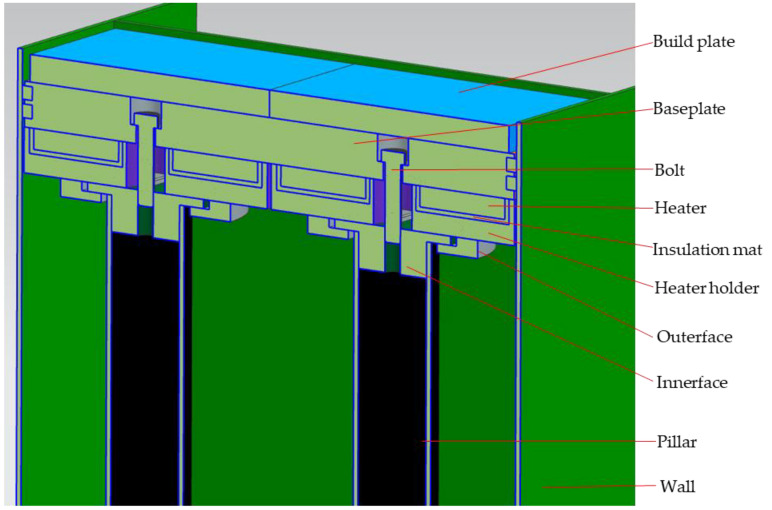
Current powder pool with preheating system.

SLM processing generates residual stresses caused by large thermal stresses due to high temperatures, laser melting, and fast cooling, resulting in potential part cracking or warping [6]. Waqar et al. [5] added that residual stresses are among the topmost common causes of premature failure of components when using SLM process, therefore implementing an in situ process technique to lower or eliminate residual stress is of high value as it will prevent parts from failing during and after the building process. Aeroswift’s current 640 × 340 × 600 mm powder bed platform features a preheating system (see Figure 2) designed to preheat the build plates (substrates) up to 600 °C to reduce residual stresses. A test conducted on the Aeroswift platform showed that the preheating system only achieves temperatures of less than 300 °C above the build plate. The stainless-steel 17-4ph powder pool (see Figure 2) contains the titanium (Ti6A14V) build plates (substrates) mounted on a mild (low-carbon) steel baseplate that rests on stainless steel 304 heater holders containing Aluminum Nitride 3 kW ceramic heaters. The heaters are isolated from the heater holders by 5 mm thick insulation mats. The stainless steel 304 heater holders rest on the stainless steel 304 inner and outer faces connected to stainless steel 304 pillars and the baseplate is bolted to the pillars through the inner face component using M20 mild (low carbon) steel bolts.

Studies have shown that using a preheating system minimizes tensile residual stresses [6,7,8,9,10,11,12], therefore improving the quality of the part and reducing part premature failure during building. A 510 Watt Nd-YAG laser was used by Liu et al. [6] to preheat yttria-stabilized zirconia ceramics to a maximum temperature of 2500 °C and observe a reduction in vertical cracks and porosity. Ali et al. [7] studied the effects of baseplate preheating on the residual stress formation, microstructure, and mechanical properties of Ti6A14V SLM-built parts using up to 800 °C baseplate preheating. They reduced residual stresses by 88.3% when preheating at 470 °C; furthermore, no substantial stresses were formed when preheating at 570 °C, 670 °C, and 770 °C. Roehling et al. [8] used an in situ annealing approach to reduce residual stresses in 316L steel bridges fabricated by LPBF using four laser diodes as preheating devices with 1.25 kW power each and observed that preheating at 625 °C substantially lowered residual stresses. Fries et al. [9] built crack-free tool inserts of WC-17Co at 900 °C baseplate preheating using an inductive heating device. They obtained microstructural and mechanical properties comparable to tools produced through conventional methods. Crack-free y-tiAl parts were built at 800 °C preheating by Calprio et al. [10], using an induction coil that heated the entire build volume. Saewe et al. [11] employed a baseplate preheating method that could heat up to 800 °C to process high special steel (HSS) and built crack-free samples when using 200 °C and 500 °C preheating temperatures. Meterns et al. [12] investigated the effects of elevated temperatures on the microstructure and mechanical properties of H13 tool steel, where a 300 W Yb:YAG fiber laser and baseplate preheating were utilized. Parts preheated at 400 °C had tensile strengths comparable to conventionally manufactured parts. Hardness was also improved over conventional materials. 

Studies on the development of baseplate preheating are limited and preheating for large volumes close to that of the Aeroswift platform has yet to be reported. Studies reviewed had lower build volumes compared to that of the Aeroswift platform, but they had higher heating power compared to the Aeroswift platform since it utilizes 6 kW to heat its current build volume. Calprio et al. [10] used 960 W to heat a 38 mm diameter volume to 800 °C, while Das et al. [13] used 20 kW to heat the entire chamber of volume. The research aimed to determine the cause of the preheating system’s inefficiency and to provide recommendations on design upgrades to achieve the desired performance. Determining the minimum insulation thickness and type of insulation was also covered in this study. Thermal analysis was performed on the current preheating system where the test was replicated in the thermal analysis to study the cause of its inefficiency. Furthermore, experimental work was carried out to test the performance of the preheating system’s components. FEA and experimental results led to the development of two preheating concepts that were also thermally analyzed using FEA. 

## 2. Materials and Methods

### 2.1. Materials

The material properties used during simulation or experimental testing are provided. Stainless steel 304 material with a density of 8000 kg/m^3^, a specific heat capacity of 504 J/kgK, and thermal conductivity of 16.2 W/mK was used in the experiments and also assigned to the heater holder, faceplate, pillars, and inner and outer faceplate during FEA. A density of 7850 kg/m^3^, a specific heat capacity of 510.8 J/kgK, and thermal conductivity of 54 W/mK were assigned to the mild steel baseplate and the bolts. The build plates in FEA were assigned titanium Ti64-Al 4 V material with a density and thermal conductivity of 4420 kg/m^3^ and 7.2 W/mK, respectively. The material (Aluminum Nitride) that forms the 290 × 290 × 23 mm 3 kW ceramic heater used in FEA and experiments had a density, specific heat capacity, and thermal conductivity of 1900 kg/m^3^, 740 J/kgK, and 180 W/mK, respectively. The FEA and insulation used a 5 mm thick Cermex BK2100SW (Mid-Mountain Materials, Inc., Seattle, WA, USA) insulation mat with a density, specific heat capacity, and thermal conductivity of 128 kg/m^3^, 700 J/kgK, and 0.11 W/mK, respectively. The insulation mat was the material used to thermally isolate the heater from the stainless steel 304 heater holder. The insulation box used for experiments was fabricated using 25 mm thick Monolux-800 fiberboards (Promat Monolux, Midrand, South Africa) with a density of 950 kg/m^3^, a specific heat capacity of 1030 J/kgK, and thermal conductivity of 0.25 W/mK. The Monolux-800 fiberboards were also used as insulation below the heater during experiments and were also used during FEA analysis as a potential insulation material. The walls of the powder pool in FEA were assigned stainless steel 17-4ph material with a density of 7750 kg/m^3^ and thermal conductivity of 17.9 W/mK. The QG-1.1 silica insulation brick with a density of 1100 kg/m^3^ and thermal conductivity of 0.6 W/mK was used in FEA as potential thermal insulation for the heater. FEA used emissivities of 0.35, 0.51, 075, 0.85, and 0.8 on all exposed surfaces of stainless steel 304 and 17-4ph, titanium Ti64-Al 4V, Monolux-800 fiberboards, and QG-1.1 silica insulation brick, respectively.

### 2.2. Methods of Experiments

**Figure 3:**
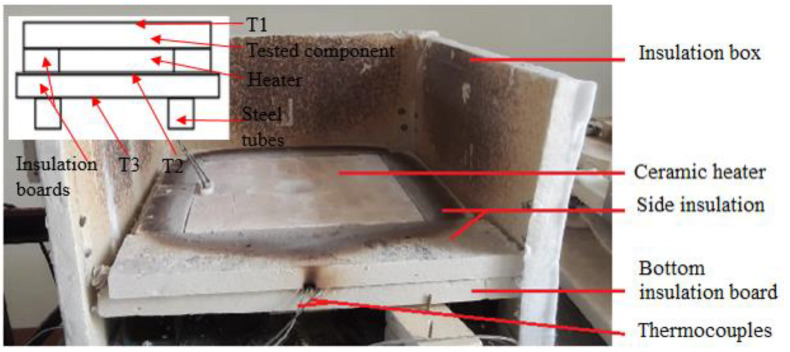
Insulation box with heater (open).

The experiments were conducted in an insulation box (see Figure 3) with a ceramic heater, which was placed above a rigid Monolux-800 insulation fiberboard supported by two 70 × 70 × 300 mm mild steel tubes. The sides of the heaters were also insulated by 70 mm wide Monolux-800 insulation fiberboards of 25 mm thickness. The heater’s resistive wires were extended to the outside of the insulation box for electrical connections. Material that had to be tested was always placed above the heater during testing. Tests were conducted with the box covered on all sides using the same material.

An outsourced power box was used to supply power to the heater, and it was equipped with a temperature control system that controlled and monitored the temperature of the heater. Three K-type thermocouples were used to measure temperatures that would be logged using an Arduino board and software. A fourth K-type thermocouple (RS Pro, Midrand, South Africa) was used by the power box to measure the temperature of the heater for control. Temperature data were acquired using an Arduino program developed in Arduino software, where three K-type thermocouples provided temperatures to a MAX6675 module, which digitized signals from the K-type thermocouples and sent them to an Arduino board, which then output the temperature data. During testing, the heater was always heated from room temperatures to targeted temperatures, where heating involved ramping the heater up at 100 °C increments with a 10 min delay between each increment until reaching the target temperature (to prevent the heater from cracking due to potential moisture).

#### 2.2.1. Heater Test

The heater specification states a temperature of 1000 °C is possible, therefore it was tested to validate the performance and also to ensure that the heater was not defective. The heater was placed above a fiberboard that rested on two mild steel 70 × 70 × 300 mm tubes, as shown in the schematic of Figure 3. Another fiberboard was placed above the heater to reduce or eliminate heat loss through radiation and convection on the top surface of the heater. A thermocouple 1 (T1) measured the temperature after (above) the top fiberboard insulation. Thermocouple 2 (T2) measured the temperature below the heater while thermocouple 3 (T3) measured the temperature below the bottom insulation, which was 50 mm thick. The heater was heated to just over 800 °C before ending the test, which took 90 min.

#### 2.2.2. Current Insulation Material Experimental Methods

This test was conducted to determine the performance of the 5 mm thick Cermex BK2100SW (Mid-Mountain Materials, Inc., Seattle, WA, USA) insulation material that was used in the Aeroswift machine to isolate the heater from the mechanical components. The insulation was placed above the heater and thermocouple 1 (T1) measured the temperature after (above) the Cermex BK2100SW insulation mat, as shown in the schematic of Figure 3 above. Thermocouple 2 (T2) measured the temperature below the heater, while thermocouple 3 (T3) measured the temperature below the bottom insulation, which were two 25 mm thick insulation boards. The heater was heated to a maximum and constant temperature of 500 °C, which was kept constant for 30 min before ending the experiment after 82 min.

#### 2.2.3. Steel Test

This experiment aimed to determine the maximum temperature above a 320 × 315 × 20 mm stainless steel plate placed above the heater. This would provide insight into the duration it takes to reach a stable and maximum temperature above the steel plate. This would also provide insight into the challenges involved in heating thick metals to high temperatures above 500 °C. The test followed the same procedures used when conducting the insulation test, the main difference was that the steel plate was placed above the heater and the heater was heated to a high temperature of 700 °C and kept constant for 35 min until being heated to a maximum and constant temperature of 800 °C for 40 min. The experiment was conducted for 184 min (3 h).

#### 2.2.4. Validation Model Experimental Methods

**Figure 4:**
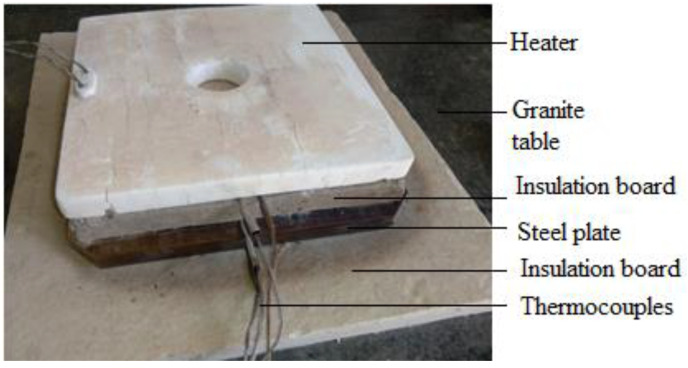
Validation model experiment.

To verify the simulation results, a basic model was created, on which thermal analysis and experimental testing were conducted to demonstrate the accuracy of the thermal analysis approach used. The experimental setup is shown in Figure 4 below, where the heater was heated to 600 °C and maintained at that temperature for three hours. Under the heater were two thermocouples: thermocouple 1 (T1) was used to record and log temperature data, and the other thermocouple was utilized by the power supply to regulate the heater’s temperature. The temperature below the insulation board was measured by thermocouple 2 (T2). Temperature below the stainless-steel plate was measured by thermocouple 3 (T3).

### 2.3. Finite Element Analysis Study

In this research work, a thermal analysis was performed using Siemens SimCenter (Thermal) solver (Siemens, Pretoria, South Africa) and the important details are given in the following subsections. FEA was performed on the current preheating system, the preheating concepts developed to improve preheating performance, the basic model developed to validate simulation results, and also the current insulation material used in the Aeroswift platform.

#### 2.3.1. Current Preheating System

Steady-state thermal analysis was conducted to replicate the test where the inefficiency of the preheating system was initially discovered, to compare results and then determine the cause of heat loss in the system. Most 3D components were defined with 3D tetrahedral meshes where TET4 element types were used for meshing. Thin large 3D components were meshed using 2D mesh, where QUAD4 thin shell element type was applied to the large surface areas and then projected to the entire thickness using the 3D swept mesh with HEXA8 element type. Mesh element sizes were first determined based on the recommended sizes by the software and then mesh convergence was performed after obtaining results to determine whether the results would change after decreasing mesh sizes by half. Material properties detailed above were used when performing the simulation. 

##### Boundary Conditions and Loads

Perfect contact resistances were assumed for all surfaces that were in contact. An ambient temperature of 30 °C and radiation losses were applied on all exposed surfaces. A heat load of 1,785,714 W/m^3^ obtained from Equation (1) was applied to the volume of the heater, where $Q^{\prime \prime }$ and *V* was the heater output power (W) and the volume (m^3^) of the heater, respectively.

(1) $$Q^{\prime \prime \prime }=\frac{Q^{\prime \prime }}{V}$$

A velocity of 1 m/s for forced convection inside the chamber was assumed based on 10 m/s obtained from Equation (2), which calculated argon circulated in the Aeroswift platform using a roots blower.

(2) Q=vA

where Q, v, and A were flow rate (m^3^/s) in a 100 mm pipe, gas velocity (m/s), and the area (m^2^) of the pipe. 

#### 2.3.2. Preheating Concept 1 (QuickPre) and Concept 2 (HeatGenPro)

Two preheating concepts were developed to achieve the target temperatures of up to 600 °C. The concepts were both thermally analyzed to evaluate the capability of reaching the target temperature. Preheating Concept 1, also referred to as QuickPre, followed a similar process to the one used during the analysis of the current preheating system. The main difference was the heat load applied, since the simulation was conducted assuming that the heater was able to produce a constant temperature of 800 °C above the heater. In Preheating Concept 2, also referred to as HeatGenPro, the main difference from the procedures used in concept 1 was that emissivity of 0.8 was used on all the exposed fire brick walls. FEA was performed with the following assumptions: steady-state, perfect contact, forced convection with a velocity of 1 m/s, and the bottom of the pillars was at 25 °C.

#### 2.3.3. Validation Model FEA Methods

**Figure 5:**
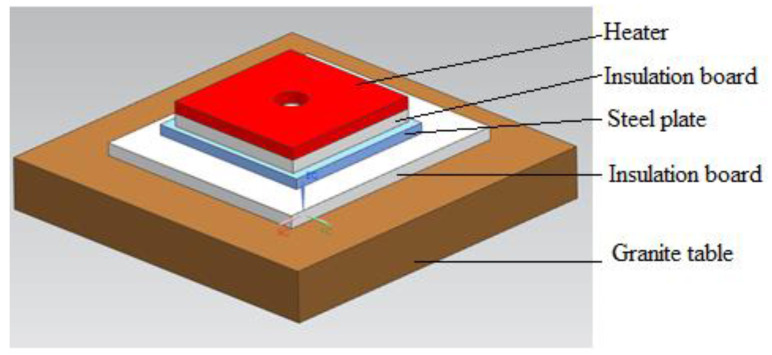
Validation model simulation.

The basic model (see Figure 5) which served as a baseline for evaluating the accuracy of the simulation work was simulated to compare the simulated and experimental outcomes. The model was meshed using a 3D tetrahedral mesh of TET4 element types and thermally analyzed using steady-state analysis. All exposed surfaces received radiation according to the emissivity of the various materials. All exposed surfaces were subjected to free convection at a 20 °C ambient temperature. A load of 600 °C was applied to the bottom surface of the heater and all surfaces in contact were assumed to be in perfect contact.

#### 2.3.4. Current Insulation Material FEA Methods

A transient state analysis was performed to simulate the experiment conducted in the insulation box to compare the results. The analysis used a similar approach to all previous analyses, where 3D tetrahedral meshes were applied to all 3D parts. Radiation and convection were applied to all exposed surfaces together with an ambient temperature of 25 °C. Surfaces in contact were assumed to be in perfect contact. To produce a maximum temperature close to 500 °C, as in the experiment, only 52% of the total 3 kW heater power was used, which was 928,571 W/m^3^ on the volume of the heater.

## 3. Results and Discussion

### 3.1. Experimental Results

#### 3.1.1. Heater Test

**Figure 6:**
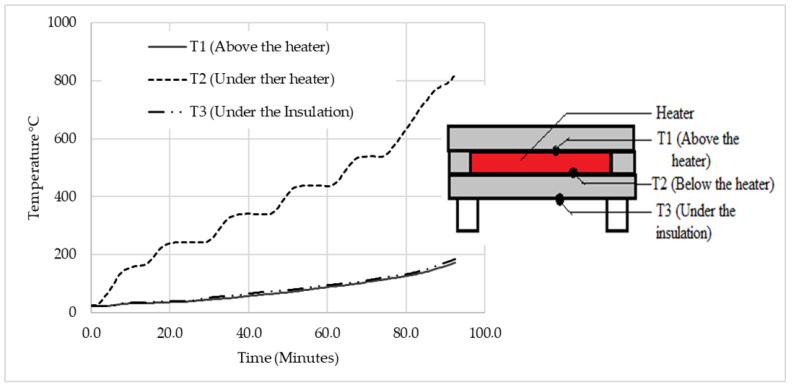
Heater thermal efficiency experimental results.

The thermal efficiency of the heater was also tested to rule it out as a possible factor that caused the preheating’s inefficiency. The heater reached 819.3 °C (see Figure 6), making it clear that it was not defective. The temperatures after the insulation (T1 and T3) were almost the same for the entire duration of the experiment since they were the same material. 

#### 3.1.2. Current Insulation Material Experimental Results

**Figure 7:**
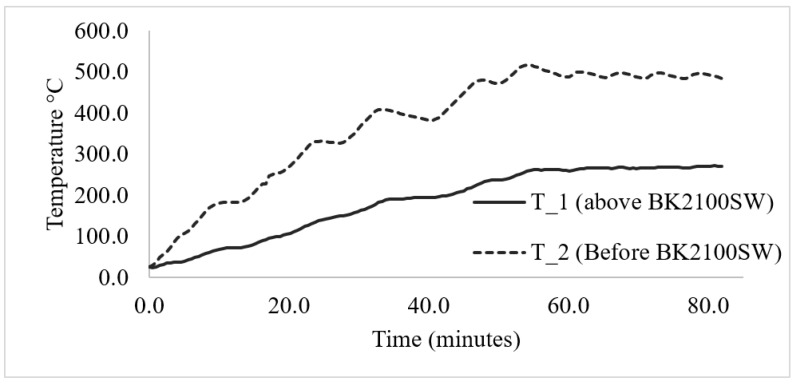
Cermex BK2100SW insulation mat temperature results.

The performance of the 5 mm thickness Cermex BK2100SW insulation mat is shown in Figure 7 below, where it can be seen that the heater was heated from room temperature to 500 °C and then kept constant for 30 min. A maximum constant temperature of 265 °C was obtained after the insulation, which shows that even for a relatively thin layer thickness, insulation is provided.

**Figure 8:**
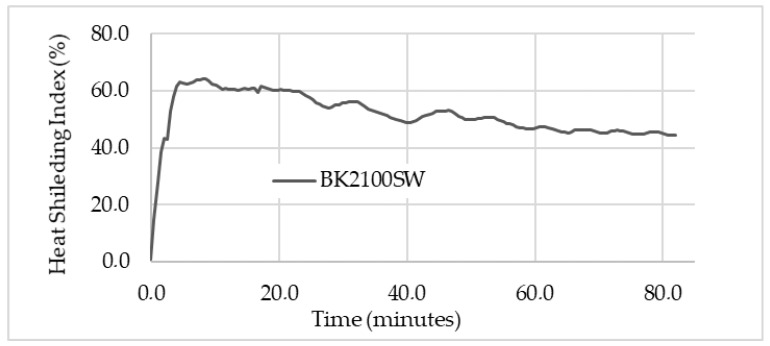
Heat shielding Index (performance) of Cermex BK2100SW insulation mat.

The performance of the Cermex KB2100SW insulation mat is illustrated in Figure 8 below using the heat shielding index (HSI). The HSI is used to illustrate the efficiency of insulation material as a percentage: a higher percentage indicates a higher efficiency and vice versa. The HSI was based on the temperature before (heated surface) and after insulation: the higher the difference, the higher the HSI, which translates to higher efficiency. The insulation mat reached a max of 64% efficiency in the beginning, as the temperature after the insulation was increased slowly due to low thermal conductivity compared to that of the heater. Initially, the heater quickly reached over 150 °C in 10 min, then the temperature after the insulation gradually increased but at a lower rate than the heater, therefore making the HSI as high as 64%. As the temperature increased over time, the performance of the insulation decreased as the temperature increased. The HSI went from 64% to 44.3%, which was not suitable, to significantly reduced heat loss in other components. Equation (3) below explains how insulation material protects against heat [14].

(3) $$\Psi =\left(\frac{T_{h}-T_{i}}{T_{\;h}}\right)\times 100\%$$

where Ψ, $T_{h}$ and $T_{i}$ are the HSI (%), the heater, and insulation temperatures (°C), respectively, and Equation (3) can be used to construct a graph that shows the performance of the insulation material over the heating duration, as seen in Figure 8 below. The HSI starts at a higher percentage because the temperature difference between the heated surface (before insulation) and after the insulation surface was high because the temperature after the insulation was still rising. When the temperature difference increased, the HSI started to decrease.

#### 3.1.3. Steel Test

**Figure 9:**
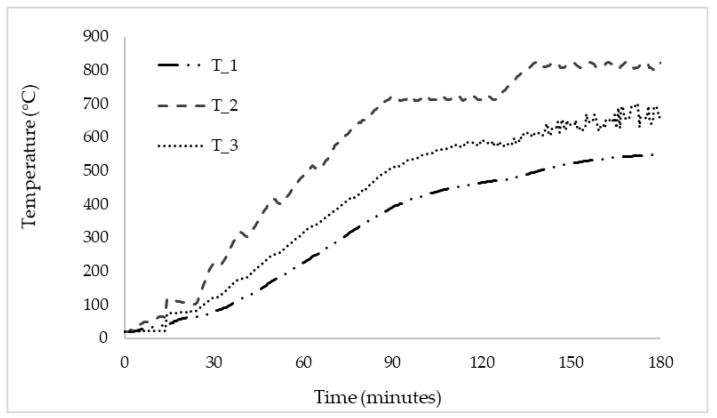
Steel plate test results.

This test was conducted to establish the maximum surface temperature above a stainless-steel plate placed on a heater at 800 °C below its surface. Duration to reach the maximum temperature above the plate was also assessed. The heater reached 800 °C after 106 min (see Figure 9) and the steel plate temperature started to stabilize at 540 °C after 180 min, where it was noticed that the temperature of the plate was struggling to rise. The maximum temperature reached after four hours of heating was 548.8 °C and 691 °C above and below the plate, respectively, with a difference of 142.2 °C between the two temperatures. After an hour of rising from 523 °C to 549 °C (a 26 °C increase), the experiment was stopped as it was noticed that no significant increase was to occur. The experiment illustrated that it was challenging to reach the target temperature due to heat loss to metals. The presence of several metals in the preheating system caused significant heat loss in the system. The test illustrated that the time taken to obtain a stable and maximum temperature above the steel plate would not be less than four hours. This experiment demonstrated the challenges experienced by the preheating system, as it was challenging to reach 600 °C with a single steel plate placed above the heater and two 25 mm thick insulation boards below it. It would therefore be a challenge to reach 600 °C in the process chamber with other metals that act as heatsinks and a powder bed that stays open to the atmosphere.

#### 3.1.4. Validation Model Experimental Results

**Figure 10:**
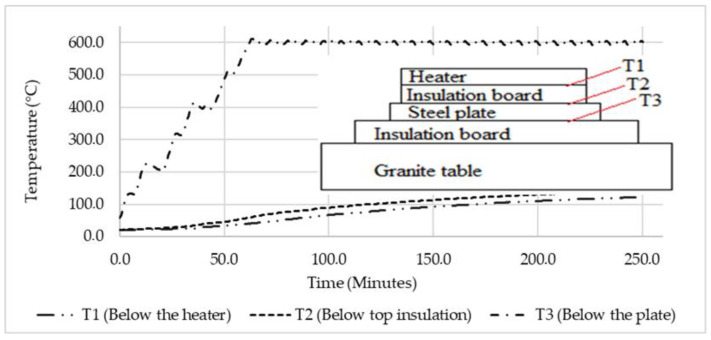
Validation model experimental results.

The temperature findings from basic experiments are illustrated in Figure 10, where the heater was heated to 600 °C and maintained at that temperature for the remainder of the experiment. After an hour, the heater reached 600 °C and maintained that temperature for three hours. After one hour, with the heater at 600 °C, the temperatures of the steel plate and insulation started to rise noticeably. Only a minor (20 °C) temperature difference existed between the steel plate and the temperature below the insulation. After 4 h of heating, the highest temperatures that could be attained below the steel and insulation were 141 °C and 122 °C, respectively.

The outcomes of the experiment and the simulation differed significantly. This happened as a result of numerous assumptions required for the FEA simulations. Convection parameters of various materials were assumed, emissivity used for radiation also affects the results if not accurate, and surface roughness affects how a material emits and reflects radiation. The simulation was carried out under the assumption that material properties would not change. The outcomes were influenced by a wide range of variables. Additionally, the simulation was run under the assumption that all surfaces had perfect contact (no heat transfer impeding).

### 3.2. Finite Element Analysis Results

#### 3.2.1. Current Preheating System

**Figure 11:**
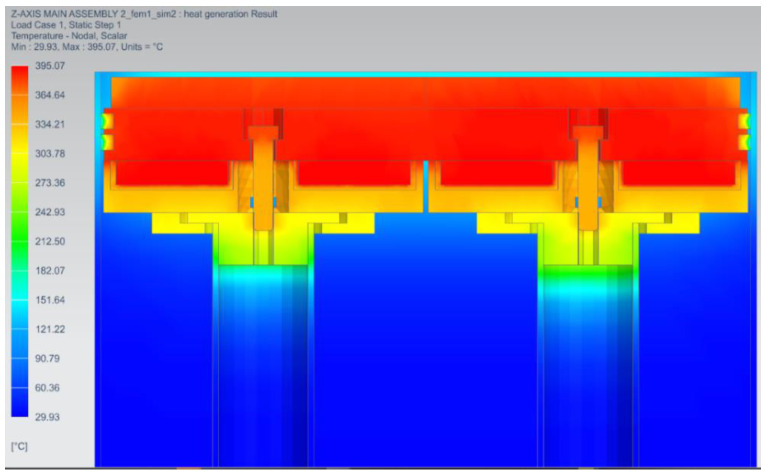
Steady-state analysis of the preheating system.

The simulation results illustrated in Figure 11 show the maximum temperatures of 395.1 °C, 390.5 °C, 374 °C, and 340 °C on the ceramic heater, baseplate, build plate, and heater holder, respectively. The build plates reached a maximum of 374 °C, which was 37.7% lower than the target temperature of 600 °C. 

The temperature of the heater holder was just 13.9% lower than that of the heater. This indicated that the 5 mm thick Cermex BK2100SW insulation failed to significantly reduce heat flow into the heater holder, although the heat was also conducted between the baseplate and the heater holder since they were directly in contact. The bolt also contributed to transferring heat from the baseplate to the inner faceplate through conduction and radiation. The bolts reached a temperature above 360 °C which was 8.8% lower than the temperature of the heater. This also showed that the bolts had high temperatures. There were several methods by which heat was transferred to metal components that caused heat loss, therefore new concepts had to be designed to reduce heat losses to metal components.

#### 3.2.2. Preheating Concept 1 (QuickPre)

**Figure 12:**
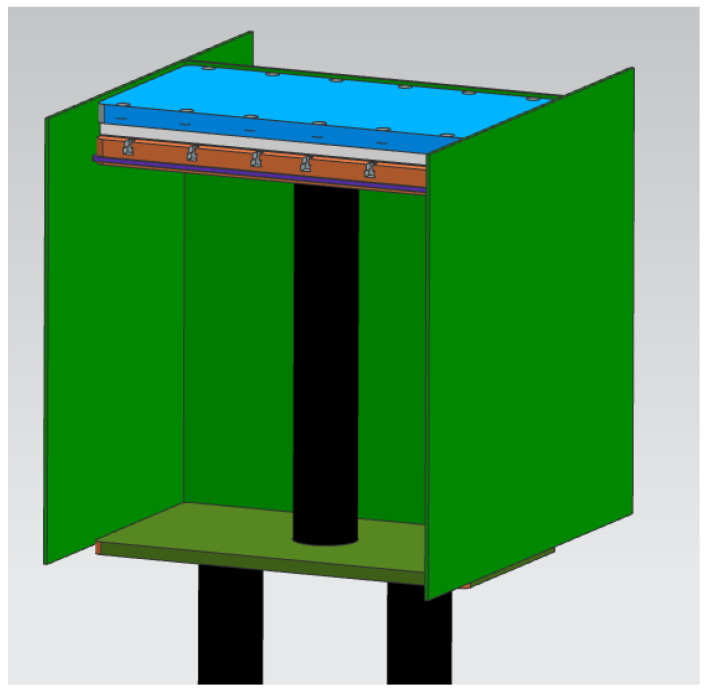
3D view of QuickPre preheating concept.

**Figure 13:**
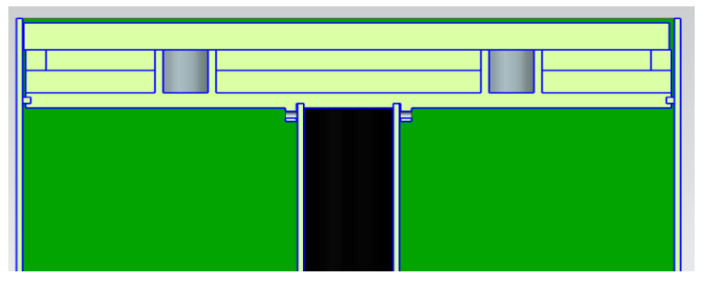
Side view of QuickPre concept.

**Figure 14:**
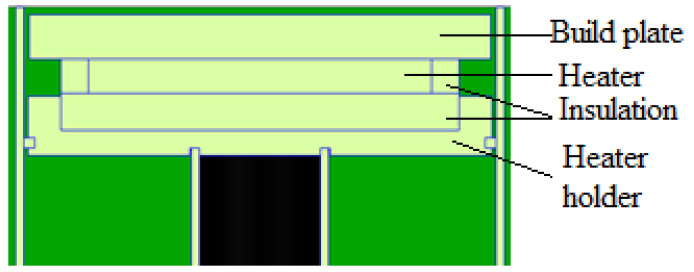
Front view of QuickPre.

Figure 12 shows a 3D view of the QuickPre concept designed for the powder pool. The concept was developed with fewer components to reduce heat loss. The baseplate, pillar, inner, bolt, and outer faceplates were eliminated, as seen in Figure 13 and Figure 14 showing all the views of the concept.

##### QuickPre Results

**Figure 15:**
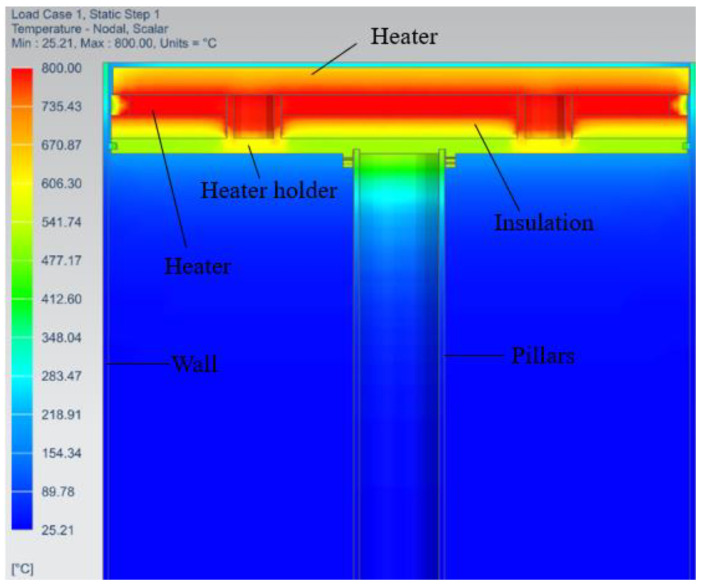
Thermal analysis results of the Concept 1 (QuickPre).

Figure 15 shows the thermal analysis results of the QuickPre concept, where the build plate reached surface temperatures of around 654 °C, illustrating better possibilities of achieving the required preheating temperatures. High temperatures of around 506 °C were observed at the heater holder; this was 36.8% lower than the heater temperature, which was an improvement from the results obtained in the FEA of the current preheating system. A lower percentage in the temperature of the heater holder compared to the heater would mean that the holder experienced high temperatures, which will cause heat loss since high temperatures are transferred to the heater holder. 

The holder still experienced a high temperature, since there was still conduction between the build plate and the holder through the support tubes, which led to the development of concept 2 (HeatGenPro). The QuickPre concept demonstrated that reducing the number of metal components such as the 50 mm thick baseplate improved the performance of the preheating system. In addition, applying a thicker insulation material has also been shown to enhance the performance of the preheating system.

#### 3.2.3. Preheating Concept 2 (HeatGenPro)

**Figure 16:**
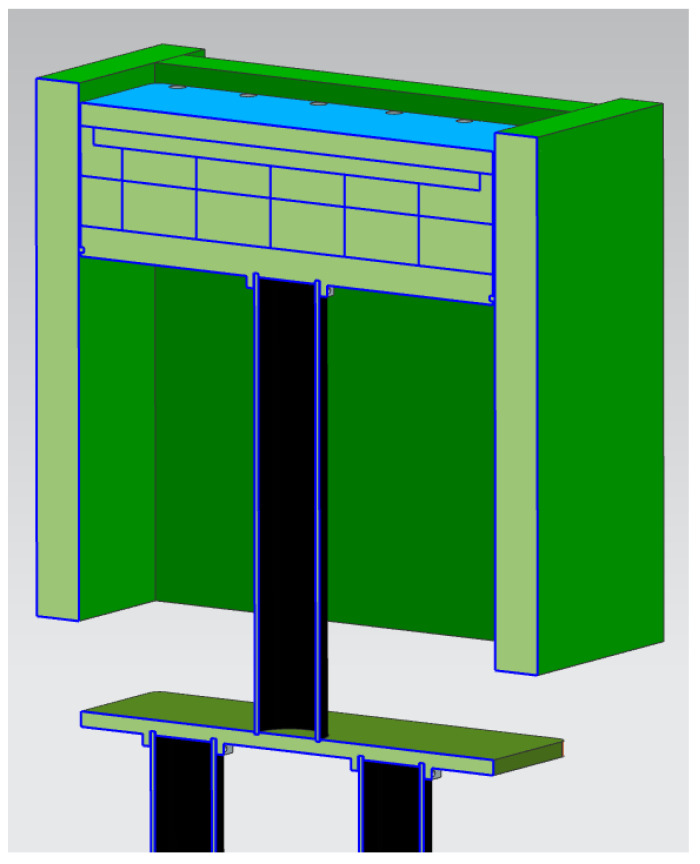
3D view of HeatGenPro preheating concept.

**Figure 17:**
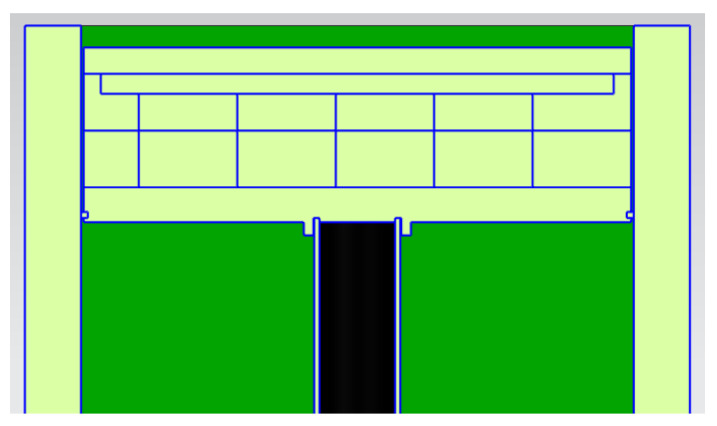
Side view of HeatGenPro concept.

**Figure 18:**
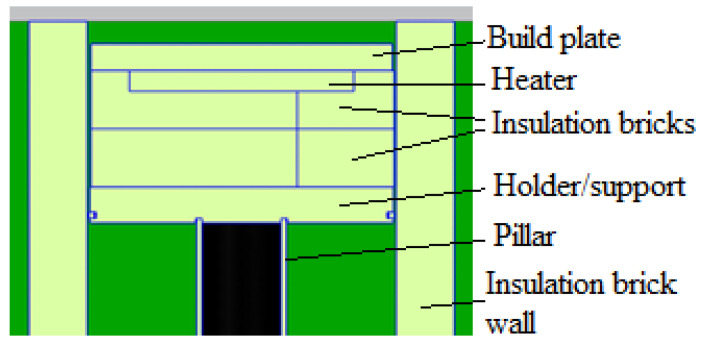
Front view of HeatGenPro concept.

The HeatGenPro concept can be seen in Figure 16 below where QG-1.1 silica insulation bricks (230 × 114 × 65 mm brick size) were used on the walls and also below the heater due to low thermal conductivities, and low thermal expansion. Additionally, QG-1.1 silica insulation bricks were selected due to their strength capability to support titanium powder and build plates as opposed to fiberboards that may deform due to the load. A machined pocket was designed in the first layer of the fire bricks to reduce heat loss on the side of the heater. The HeatGenPro concept was designed to be insulated by a minimum thickness of 107 mm of fire bricks below the heater. One layer of fire bricks was also used on the walls where stainless steel 17-4 ph was previously used. This was carried out to reduce the loss caused by convection and radiation. Side and front views of the HeateGenPro concept are shown in Figure 17 and Figure 18.

##### HeatGenPro Results

**Figure 19:**
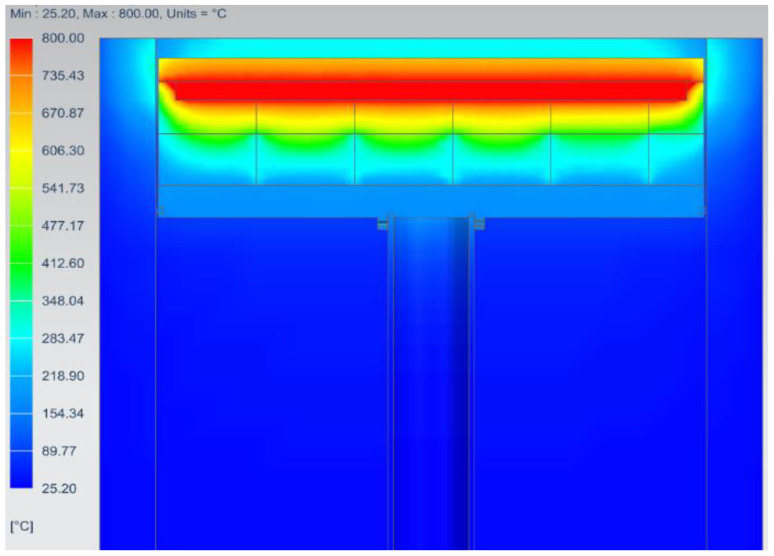
Thermal analysis results of the HeatGenPro Concept.

Figure 19 shows that the build plate reached temperatures of around 678 °C, making it 13% higher than the required 600 °C. This illustrates higher possibilities of achieving the required temperature above the build plate. The QG-1.1 silica insulation brick insulation material was seen to reduce the heat loss to the holder, with low temperatures of around 168 °C, which was 63.5% lower than 460 °C obtained in the QuickPre. The elimination of contact between the heater and metal object together with increasing the thickness of the insulation material below the heater was seen to improve the performance of the preheating system.

#### 3.2.4. Comparison of Preheating Systems 

Table 1 below compares the different features of the various designs of preheating systems from the current system to the developed concepts. The comparisons are based on design models and simulation results.

#### 3.2.5. Validation Model FEA Results

**Figure 20:**
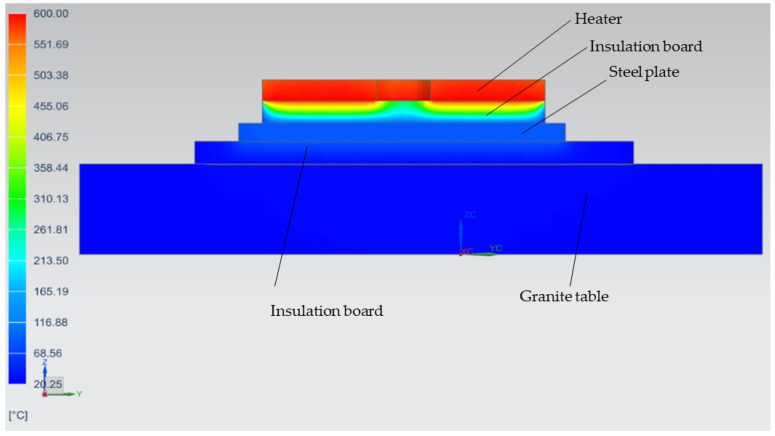
Validation model simulation (FEA) results.

The outcomes of the thermal analysis are displayed in Figure 20 below. The temperature was roughly 84 °C under the insulation, whereas under the steel plate, it was roughly 80 °C when the heater was heated to a constant temperature of 600 °C in an open-air environment.

The results of the simulation and the experiments differed between 60 °C and 75 °C. The mismatch was caused by a variety of factors, including the simulation’s use of assumptions such as convection parameters and the possibility that the emissivity values employed would not match those found in the experiment because emissivity depends on the texture of the material. Convection was possibly not constant in reality, although the simulation used constant convection parameters. Since ambient temperature fluctuates in practice, the ambient temperature utilized may be slightly different from that of the experiment. The precision of the material’s sizes and regions may also deviate slightly from reality. Additionally, the simulation was run under the assumption that the material’s properties remained constant, heat transfer between components was constant and the materials were homogeneous.

#### 3.2.6. Current Insulation Material FEA Results

**Figure 21:**
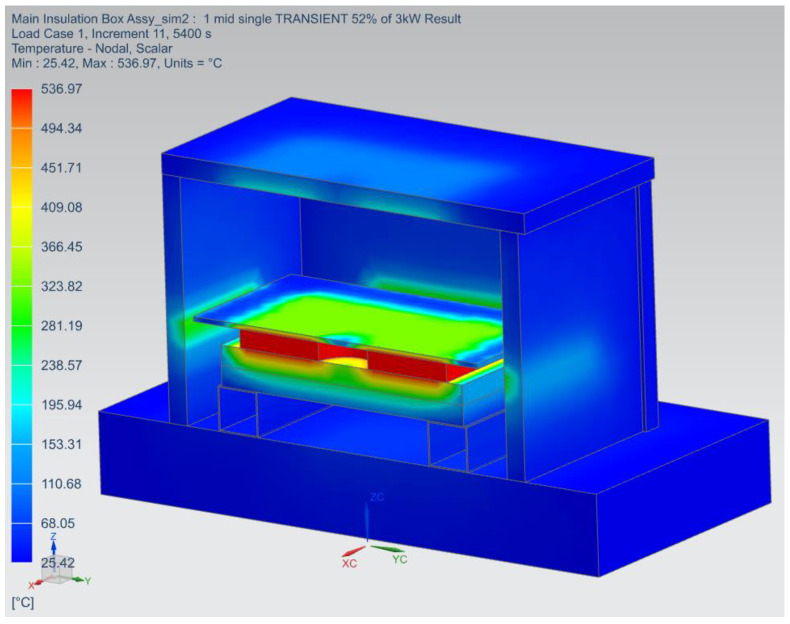
Current insulation material thermal analysis results.

Figure 21 below shows the results of the analysis performed on the Cermex BK2100SW insulation material used to thermally isolate the heater from the heater holder. The transient state was performed with a heat load of 52% to match the heater temperature used during the experiment. The simulated heater temperature reached a maximum temperature of 537 °C at 52% heater power. The temperature experienced below the insulation was also 537 °C. The temperature after the 5 mm insulation was around 319 °C, which was just 47 °C higher than the temperature obtained above the insulation during the experiment. The difference between the simulated and experimental heater temperatures was around 37 °C, which illustrated that the simulation and experimental results were comparable for practical purposes.

**Figure 22:**
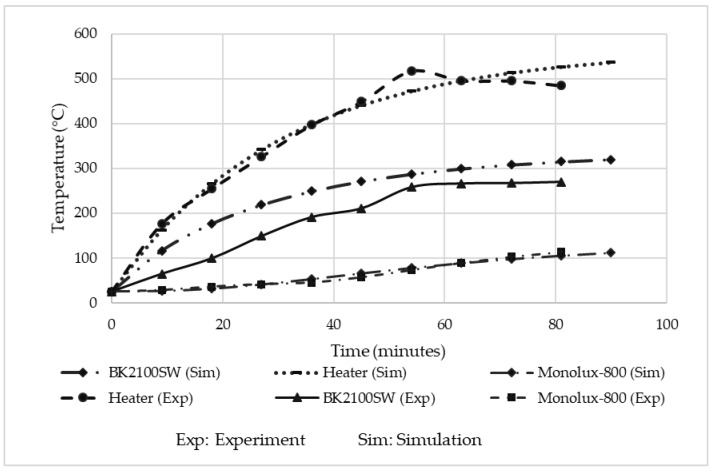
Comparison of simulated and experimental results for the current insulation material.

Figure 22 below compares the simulation and experimental results. It can be seen that the heater temperatures were in close agreement with one another for the entire duration of the test. 

The simulated heater temperature was slightly higher by 40 °C and this was because the power had to be estimated at around 52% to be able to achieve a temperature of around 500 °C on the heater. This also caused the maximum temperature above the insulation to be slightly higher in the simulation, which was 45 °C higher than the experimented temperatures. Additionally, the experimental and simulated temperatures below the Monolux-800 insulation board were very close to each other, with just a 1 °C to 3 °C difference, which illustrated that the simulated results are reasonable.

### 3.3. Numerical Study

After understanding that the lack of adequate insulation material around the heater contributed to the heat loss to the heater holder, it was necessary to establish the minimum thickness that should be adequate to significantly reduce heat loss. 

#### Minimum Insulation Thickness

**Figure 23:**
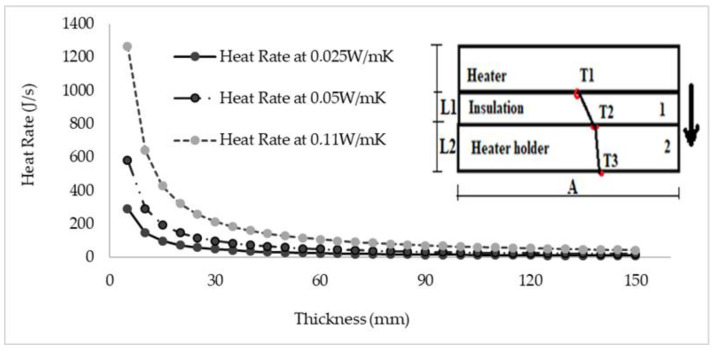
Heat rate vs. Insulation thickness at 0.025, 0.05, and 0.11 W/mK.

To establish the minimum thickness of an insulation material adequate to significantly reduce heat loss in the preheating system, a simple system (see Figure 23) was taken from the preheating system consisting of the heater, insulation, and heater holder. Assuming an adiabatic process on both sides, 1D heat transfer was considered downward. The aim was to obtain a maximum temperature of 100 °C at T3 while maintaining 800 °C below the heater; radiation and convection resistance beyond T3 was neglected. Insulation thickness was varied at three different thermal conductivities of 0.025 W/mK, 0.05 W/mK, and 0.11 W/mK to also understand the effect of thermal conductivity on heat rate, while other parameters remained constant. It was observed that to significantly reduce heat loss in the simple model, the minimum thickness should be around 25–40 mm; beyond that, it would be less effective on heat rate reduction. It can also be seen that the lowest thermal conductivity resulted in a lower heat rate, which would be advantageous when using insulation with low thermal conductivity and thickness between 20 and 40 mm. The heat flow rate (q) was calculated using Equation 4 below, where T, *L*, and *A* were temperatures (K), thicknesses (m), and Areas (m^2^) of the respective components and their surface.

(4) $$q=\frac{T_{1}-T_{3}}{\frac{L_{1}}{K_{1}A_{1}}+\frac{L_{2}}{K_{2}A_{2}}}\;\left(\frac{J}{s}\right)\;$$

It can be seen from the results in Figure 23 that using a 20 mm thick insulation material for the range of thermal conductivities evaluated (0.025–0.11 W/mK) reduces the heat rate by 75% compared to a 5 mm insulation thickness. A higher heat rate can be seen when the thermal conductivity was higher and the insulation thickness was thinner.

## 4. Conclusions

The current research studied the performance of a preheating system in AM for a large build volume powder bead system that does not perform sufficiently. The following conclusions were made from the study:

- FEA revealed that the heater holder experienced high temperatures that were only 13.9% lower than the heater, which was at 395 °C, indicating that the insulation did not significantly reduce heat flow from the heater to the heater holder.
- A test carried out to confirm the poor performance of the current insulation showed its efficiency was only 44%. This was after the back surface of the insulation reached 270 °C when its front surface was heated to 500 °C. An increase in insulation thickness is required to obtain target objectives, or an alternative material is required.
- Analytical calculations performed showed that due to significantly lower heat loss, the Cermex BK2100SW insulation material requires a minimum thickness of 20 mm.
- The QuickPre concept developed to improve the performance of the heater illustrated the capability of reaching 650 °C above the build plate during its thermal analysis. This was because several thick components that caused more heat loss were eliminated.
- Thermal analysis performed on the HeatGenPro concept showed that the build plate reached 678 °C surface temperatures, with the holder reaching around 168 °C, which was 63.5% lower than 460 °C obtained in the QuickPre concept.

HeatGenPro is recommended for fabrication and implementation and is anticipated to achieve the target temperature of 600 °C above the build plate, owing to the elimination of several mechanical components with high thermal conductivities. This study demonstrated the cause of the inefficiency of the preheating system; moreover, two ideas were constructed and simulated that demonstrated increased performance to attain elevated temperatures. It has given researchers a better understanding of the development and improvement of a high-temperature preheating system for large-scale additive manufacturing systems, which will facilitate the mitigation of adverse residual stresses that cause premature failure of parts in Additive Manufacturing, particularly Selective Laser Melting. Further research is required to establish the effectiveness of higher pre-heating temperatures to reduce residual stresses for large scale AM builds. Furthermore, the impact of applying increased temperature on titanium (Ti-6Al-4V) powder should be investigated to determine its effect on reusability.

## Figures and Tables

**Table 1:** Comparisons of preheating system designs.

Current System Features	QuickPre Concept 1 Features	HeatGenPro Concept 2 Features
2 × 3 kW heater	2 × 3 kW heater	2 × 3 kW heater
5 mm thick insulation	25 mm thick insulation	107 mm thick insulation
22 mm thick heater holder	20 mm thick heater holder base	20 mm thick heater holder base
50 mm thick baseplate	50 mm thick baseplate (eliminated)	50 mm thick baseplate (eliminated)
Outerface	Outerface (eliminated)	Outerface (eliminated)
Innerface	Innerface (eliminated)	Innerface (eliminated)
2 × build plates	2 × build plates (eliminated)	2 × build plates (eliminated)
2 × pillars	1 × pillar remained	1 × pillar remained
6 mm steel wall	6 mm steel wall	65 mm insulation brick wall
398 °C maximum temperature	650 °C maximum temperature	678 °C maximum temperature

## Data Availability

The data presented in this study are available on request from the corresponding author. The data are not publicly available due to the legal and privacy issues.
